# Supplementary material for: The biomechanical basis of biased epithelial tube elongation in lung and kidney development
Source: Development. 2021 May 4;148(9):dev194209. doi: 10.1242/dev.194209 (PMC8126414; doi:10.1242/dev.194209)
Supplement: Supplementary information [file develop-148-194209-s1.pdf]

## SUPPLEMENTARY TABLES:

**Table S1: Parameter values used in the tube collapse simulations.**

| Parameter | Description | Value | Dimension |
|---|---|---|---|
| E | Young's modulus | $10^5$ | Mass (Length)$^{-1}$ (Time)$^{-2}$ |
| ν | Poisson ratio | 0.4 | - |
| ρ | Mass density | $10^3$ | Mass (Length)$^{-3}$ |
| τ | Viscous time scale | $10^{-3}$ | Time |
| t | Epithelial thickness | $10^{-3}$ | Length |
| R | Tube radius | 2 x $10^{-3}$ | Length |

**Table S2: Parameter values used in the Chaste simulations.**

| Parameter | Description | Value | Dimension | Reference |
|---|---|---|---|---|
| η | Drag coefficient | 1.0 | Time (Length)$^{-1}$ | (Fletcher et al. 2013) |
| λ | Area deformation energy coefficient | 100 | Force (Length)$^{-3}$ | (Fletcher et al. 2013) |
| β | Membrane surface energy coefficient | 10 | Force (Length)$^{-1}$ | (Fletcher et al. 2013) |
| γ | Cell-cell adhesion energy coefficient | 0,5 | Force | (Fletcher et al. 2013) |
| T cycle | Cell cycle duration | N(12, 7.2) | Time | Figure 7 C,D |
| Ad | Area division threshold | N(1.5, 0.9) | Length$^2$ | Figure 7 C,D |
| Dt | Timestep | 0.002 | Time | (Fletcher et al. 2013) |
| d min | Cell rearrangement threshold | 0.01 | Length | (Fletcher et al. 2013) |

## SUPPLEMENTARY FIGURES

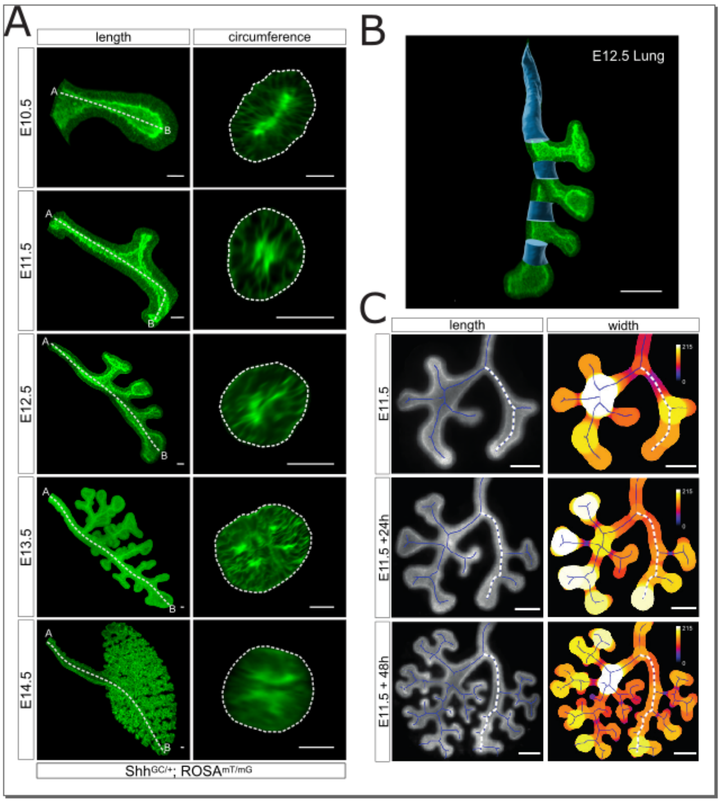


**Figure S1: Measuring epithelial morphology in growing epithelial tubes**
**(A)** 3D morphometric measurements of branch length and average circumference for a developmental timeline of a mouse lung. Specimens were serially isolated between E10.5 and E14.5 and carried the $Shh^{GC/+}$; $ROSA^{mT/mG}$ reporter, which enabled the visualization of the embryonic epithelium. Branch length was measured from below the carina to the most distal tip, while average branch circumference was calculated for tubular cross-sections. Scale bars 50 µm. **(B)** E12.5 mouse lung with iso-surface overlays denoting tubular sections used for morphometric quantifications (blue). Scale bar 200 µm. **(C)** 2D morphometric measurements of length and diameter for an E11.5 lung cultured on a filter for 48h. Width scale in µm. Scale bars 200 µm.

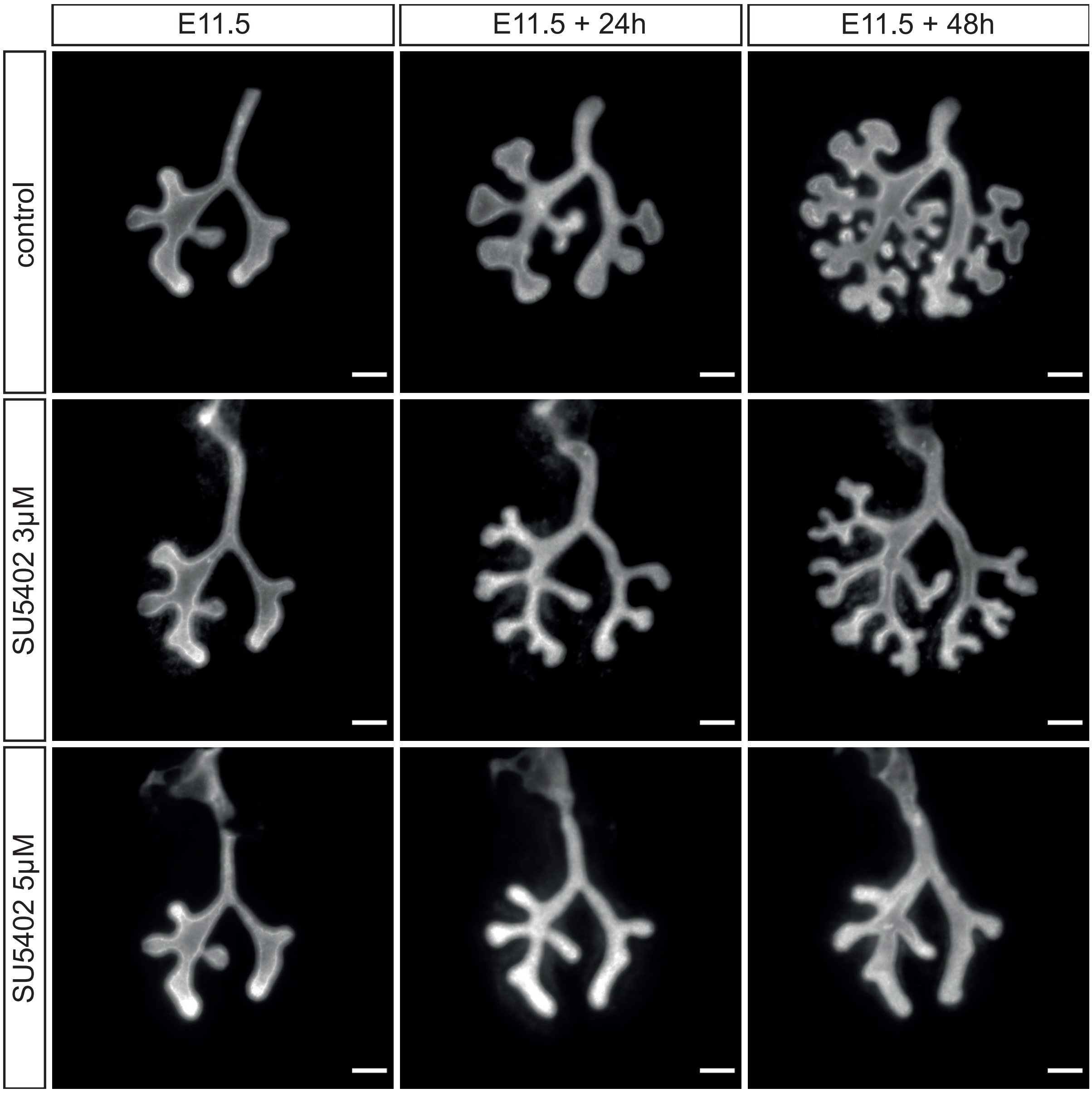


**Figure S2: Lung explant cultures with FGFR inhibitor SU5402**

Culture of E11.5 embryonic lungs under control conditions and under different concentrations of the FGFR inhibitor SU5402 for 48h. Scale bars 200 µm.



Development • Supplementary information

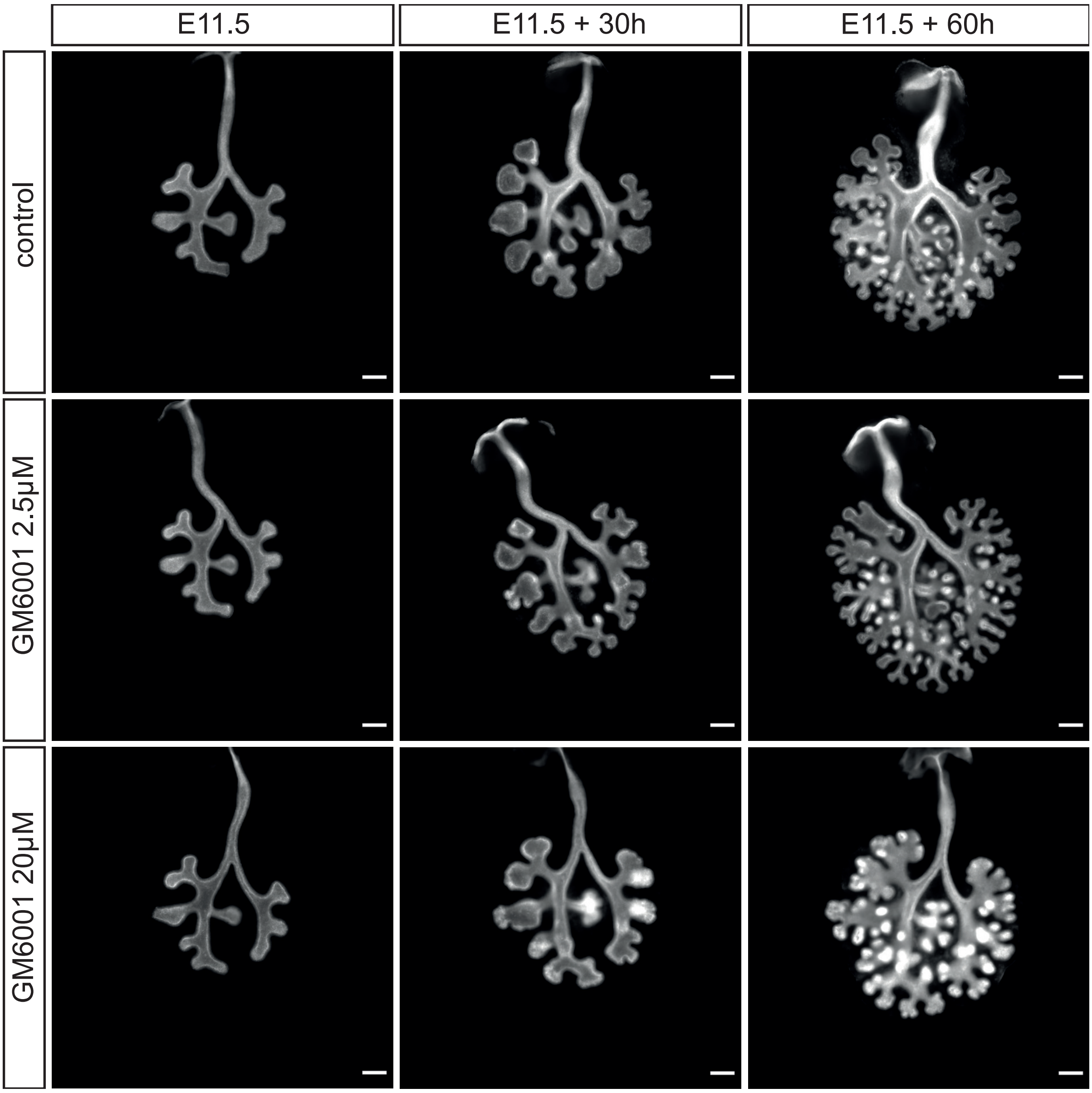


**Figure S3: Lung explant cultures with MMP inhibitor GM6001**
Culture of E11.5 embryonic lungs under control conditions and under different concentrations of the MMP inhibitor GM6001 for 60h. Scale bars 200 µm.

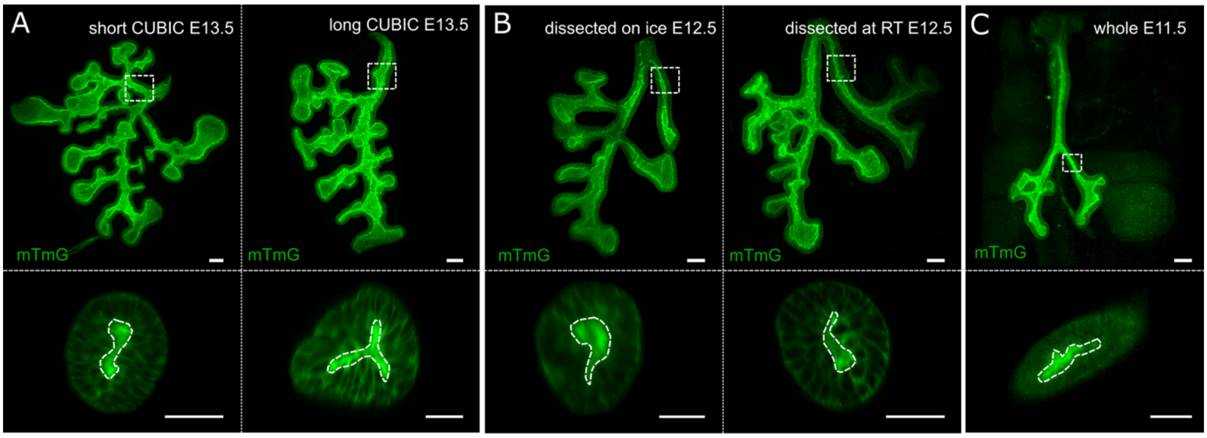


**Figure S4. Narrow luminal spaces are not the result of dissection /clearing conditions** 3D rendering of mouse lung explants (top) and cross-sectional slices (bottom) of specimens **(A)** cleared using a short CUBIC regimen (2 days in reagent-1, 2 days in reagent-2) and a long CUBIC regimen (4 days in reagent-1, 4 days in reagent-2) displaying narrow luminal spaces. Similarly, **(B)** both explants dissected on ice and at room temperature (RT), and cleared using a short CUBIC regimen, also showed collapsed lumens. Furthermore, the same luminal morphology was observed in **(C)** CUBIC cleared whole-embryos imaged through thoracic cavity. Scale bars 100 µm.

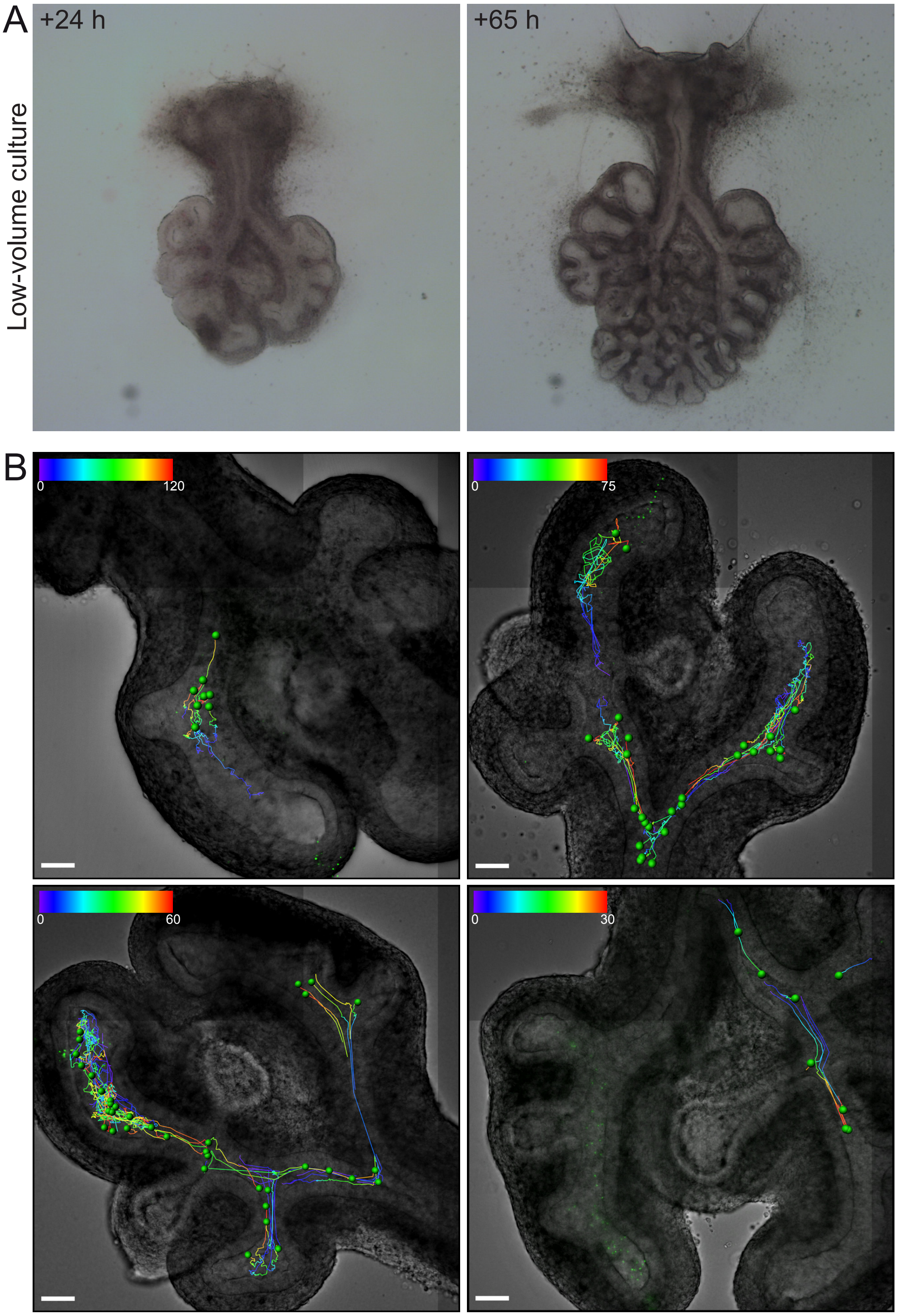


**Figure S5: Bead time-lapse imaging and tracking in low-volume cultured E11.5 lungs**
**(A)** Brightfield images of E11.5 lungs after injection and spinning disk confocal imaging after 24 h and 65 h continued low-volume culture. The culture medium contains 20% Matrigel. The liquid explant culture medium was replaced after spinning disk confocal imaging and after one day of culture. **(B)** Brightfield images of injected E11.5 lungs overlayed with the confocal bead images and the bead tracking (Imaris) of four experiments. Green dots mark the bead position at the end of the track. Colour bar denotes time (min) of tracks; scale bar 70 µm.

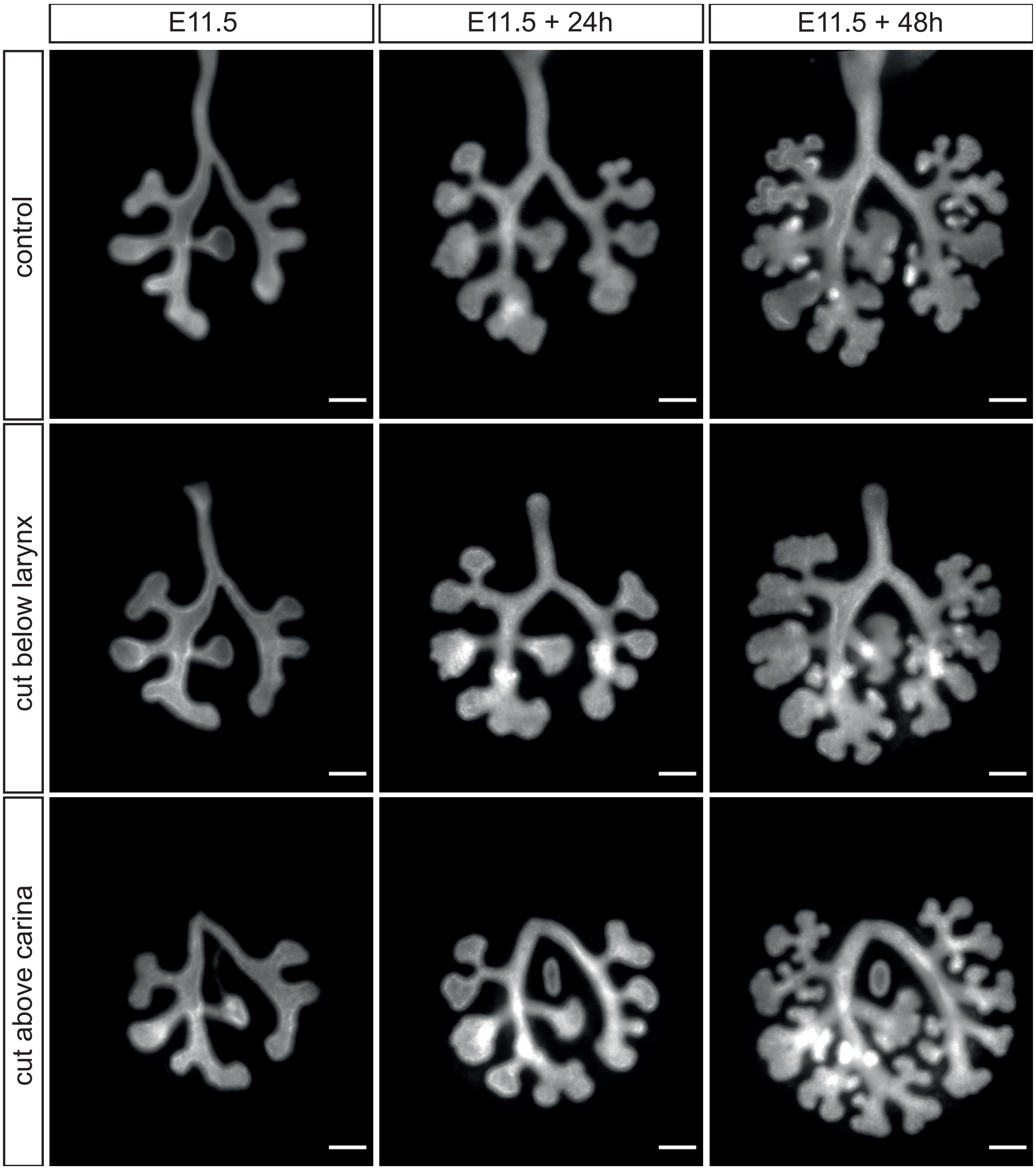


**Figure S6: Lung explant cultures with intact and cut trachea**
Culture of E11.5 embryonic lungs with intact trachea as control condition and with tracheas either cut below the larynx or above the carina for 48h. Altering the tracheal length does not impact on branching morphogenesis. Scale bars 200 μm.

Development • Supplementary information

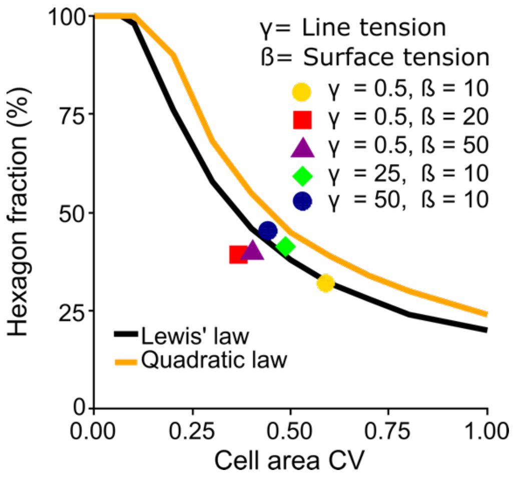


**Figure S7: Impact of parameter variations in the Chaste simulations**

Impact of the membrane surface energy β and the cell-cell adhesion energy γ on the hexagon fraction and area CV in the Chaste simulations. As expected for a tissue representation of epithelia, the simulations reproduce the relationship between the hexagon fraction and the cell area CV as predicted based on Lewis' law (black) or the quadratic law (yellow line) (Kokic et al., 2019).

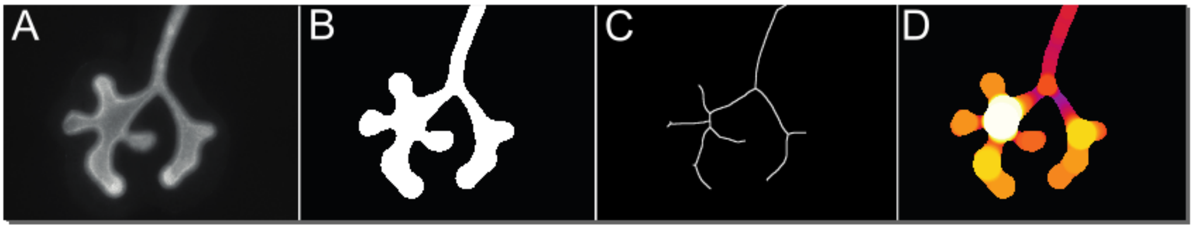


**Figure S8: Image segmentation and skeletonization**

Segmentation of raw images **(A)** resulted in binary images **(B),** which were then used to generate skeletons (**C**, inverted for illustration purposes). Thickness map images **(D)** were generated with the Fiji plugin BoneJ to infer branch widths.



Development • Supplementary information

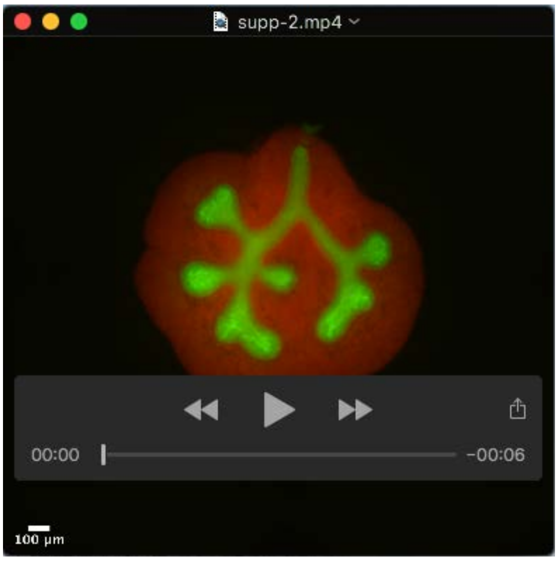


**Movie 1. Lung explant culture**
Time-lapse movie of a $Shh^{GC/+}$; $ROSA^{mT/mG}$ E11.5 lung cultured for 60h. Mesenchyme shown in red, *Shh*-expressing epithelium in green. (AVI 6.4 MB).

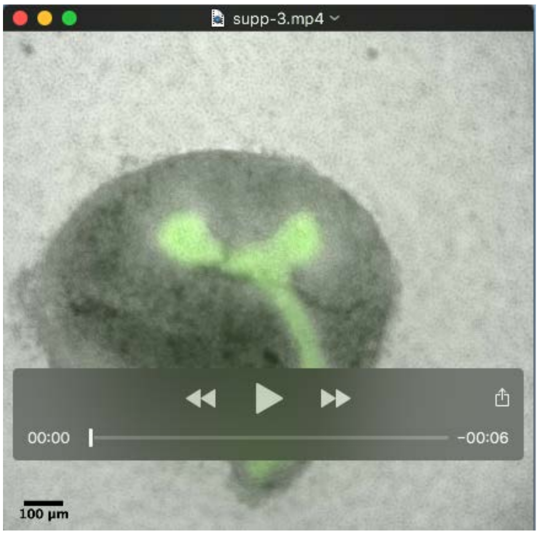


**Movie 2. Kidney explant culture**
Time-lapse movie of an E11.5 kidney cultured for 60h. HoxB7/myr-Venus expression is shown in green, brightfield in grey. (AVI 18.7 MB)

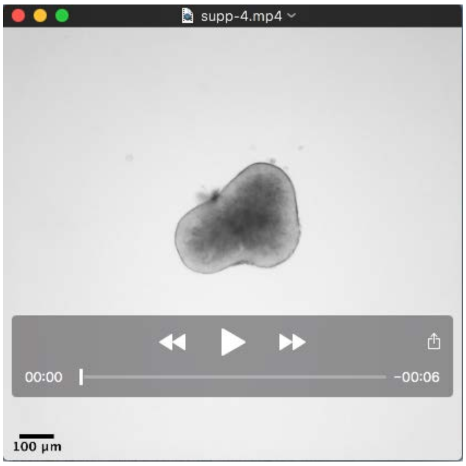


**Movie 3. Mesenchyme-free lung bud culture**
Time-lapse movie of a mesenchyme-free left lung bud dissected at E11.5 and cultured for 60h. (AVI 7.9 MB).

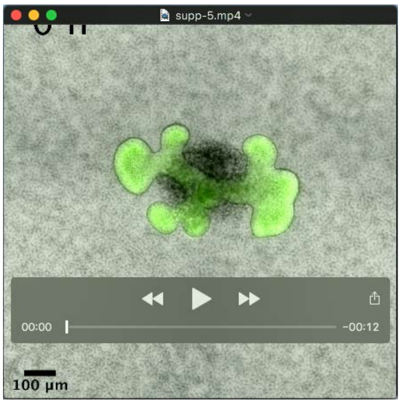


**Movie 4. Mesenchyme-free ureteric bud culture**
Time-lapse movie of a mesenchyme-free ureteric bud dissected at E11.5 and cultured for 60h. HoxB7/myr-Venus expression is shown in green, brightfield in grey. (AVI 10.8 MB).

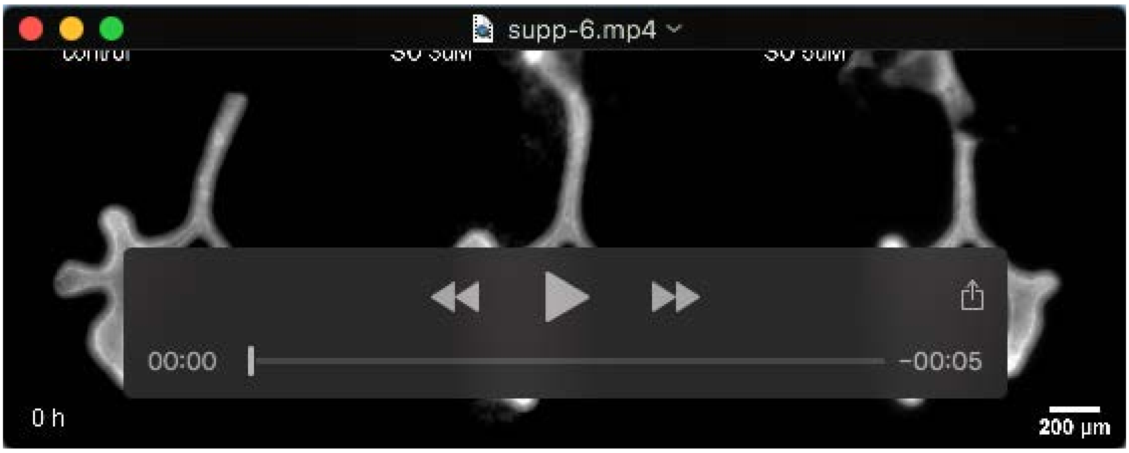


**Movie 5. Lung explant cultures with FGFR inhibitor SU5402**
Culture of embryonic lungs (E11.5) under control conditions and with the treatment of the FGFR inhibitor SU5402 at different concentrations for 48h. (AVI 8.16 MB).

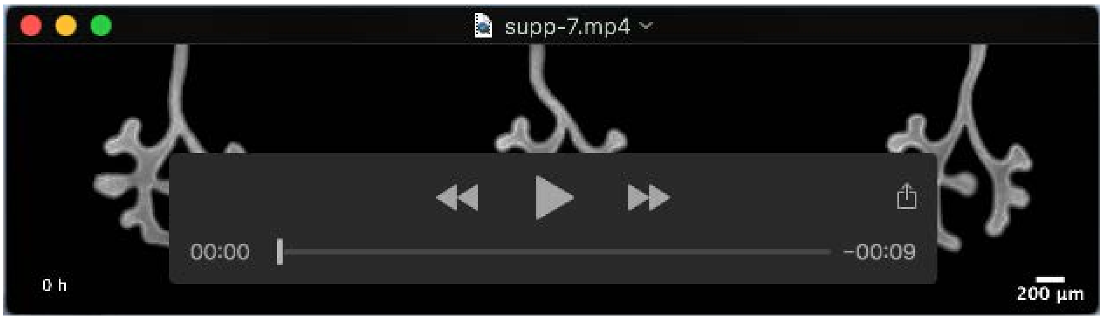


**Movie 6. Lung explant cultures with MMP inhibitor GM60001**
Culture of embryonic lungs (E11.5) under control conditions and with the treatment of the MMP inhibitor GM6001 at different concentrations for 60h. (AVI 5.3 MB).

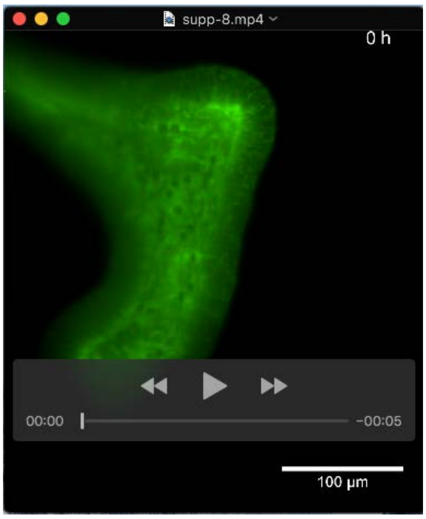


**Movie 7. High-resolution light-sheet microscopy time-lapse imaging of lung bud elongation**
Time-lapse movie showing the development of an E11.5 left lung rudiment carrying the $Shh^{GC/+}$; $ROSA^{mT/mG}$ construct. The specimen was mounted in a hollow cylinder made from low-melting-point agarose and filled with matrigel to replicate the native microenvironment and promote near-physiological growth. Imaging was done using the Zeiss Z.1 Lightsheet system for 34h. (AVI 2.38 MB).

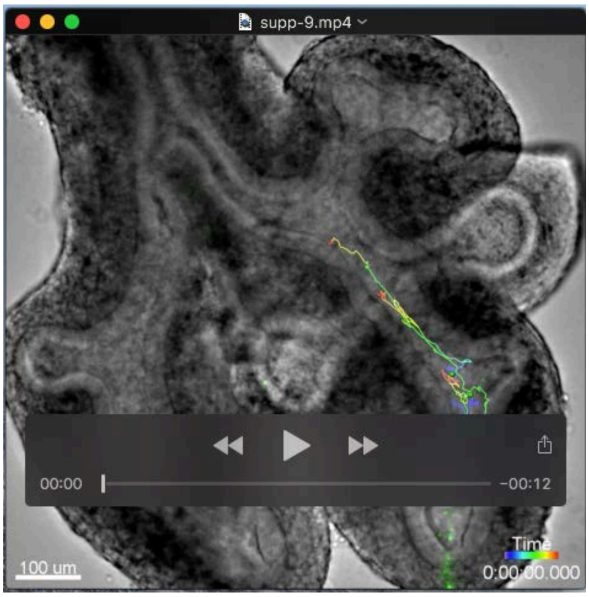


**Movie 8. Tracking of beads in the lung lumen**
Time-lapse movie of a 2 h spinning disk confocal acquisition (1 min time steps between frames) of fluorescent beads injected into the lung lumen. Bead tracks were generated in Imaris, green dots mark the detected bead at any given time point. (AVI 9.2 MB).

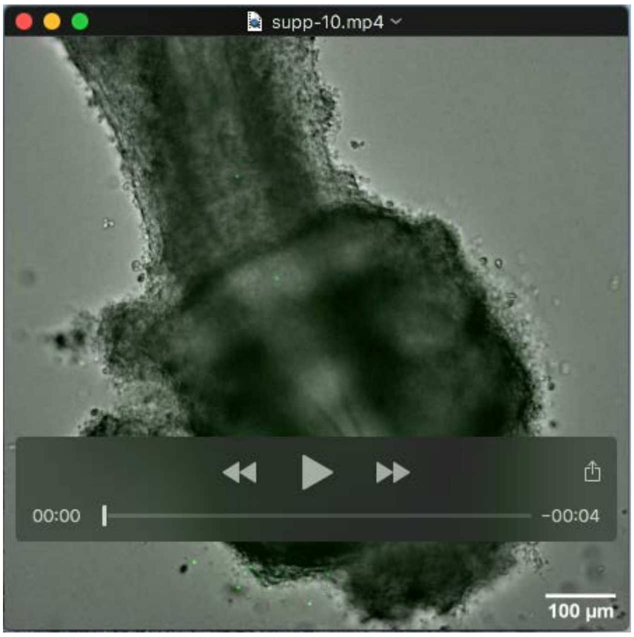


**Movie 9. Outflow of fluid from the trachea opening**
Spinning disk confocal imaging of fluorescent beads (488nm excitation channel, shown in green) overlayed with a brightfield image of the trachea of an E11.5 lung after 1h in low-volume culture. (AVI 2.5MB)

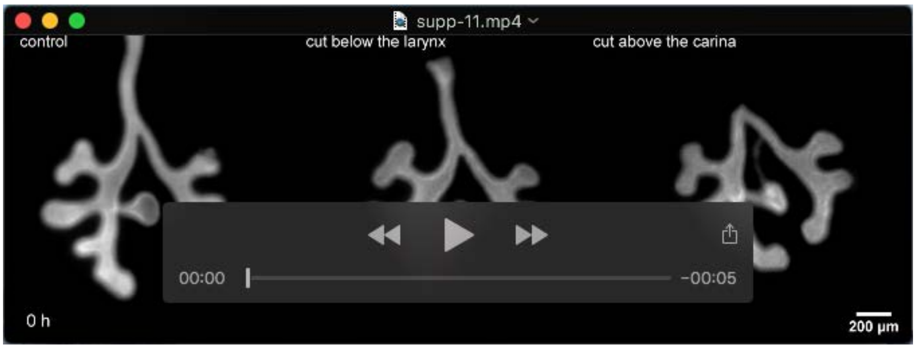


**Movie 10. Lung explant cultures with intact and cut trachea.**
Culture of E11.5 embryonic lungs with intact trachea as control condition and with tracheas either cut below the larynx or above the carina for 48h. (AVI 11.8 MB).

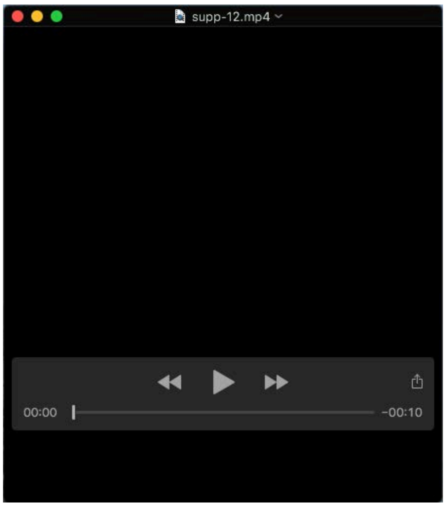


**Movie 11. Tissue simulation with external force**
Tissue growth simulation results of a vertex model when the initial configuration is subjected to a stretching force of 1.5 a.u. The parameters for the simulation are given in Supplementary Table 2.



Development • Supplementary information
