# Supplementary material for: The biomechanical basis of biased epithelial tube elongation in lung and kidney development
Source: Development. 2021 May 4;148(9):dev194209. doi: 10.1242/dev.194209 (PMC8126414; doi:10.1242/dev.194209)
Supplement: Reviewer comments [file develop-148-194209_review_history.pdf]

The biomechanical basis of biased epithelial tube elongation in lung and kidney development

Lisa Conrad, Steve Vincent Maurice Runser, Harold Fernando Gómez, Christine Michaela Lang, Mathilde Sabine Dumond, Aleksandra Sapala, Laura Schaumann, Odyssé Michos, Roman Vetter and Dagmar Iber

DOI: 10.1242/dev.194209

Editor: Paul François

Review timeline

Original submission:	22 June 2020
Editorial decision:	4 August 2020
First revision received:	1 February 2021
Editorial decision:	1 March 2021
Second revision received:	12 March 2021
Accepted:	16 March 2021

Original submission

First decision letter

MS ID#: DEVELOP/2020/194209

MS TITLE: The Biomechanical Basis of Biased Epithelial Tube Elongation

AUTHORS: Steve Runser, Lisa Conrad, Harold Gomez, Christine Lang, Mathilde Dumont, Aleksandra Sapala, Laura Kramps, Odysse Michos, Roman Vetter, and Dagmar Iber

I have now received all the referees' reports on the above manuscript, and have reached a decision. The referees' comments are appended below, or you can access them online: please go to BenchPress and click on the 'Manuscripts with Decisions' queue in the Author Area.

As you will see, the referees express considerable interest in your work, but referee 2 in particular has some significant criticisms and we therefore recommend a substantial revision of your manuscript before we can consider publication. We believe the question raised by referee 2 on the fluid flow in the lumen is particularly important to address. If you are able to revise the manuscript along the lines suggested, which may involve further experiments, I will be happy receive a revised version of the manuscript. Your revised paper will be re-reviewed by one or more of the original referees, and acceptance of your manuscript will depend on your addressing satisfactorily the reviewers' major concerns. Please also note that Development will normally permit only one round of major revision.

We are aware that you may currently be unable to access the lab to undertake experimental revisions. If it would be helpful, we encourage you to contact us to discuss your revision in greater detail. Please send us a point-by-point response indicating where you are able to address concerns raised (either experimentally or by changes to the text) and where you will not be able to do so within the normal timeframe of a revision. We will then provide further guidance. Please also note that we are happy to extend revision timeframes as necessary.

Please attend to all of the reviewers' comments and ensure that you clearly highlight all changes made in the revised manuscript. Please avoid using 'Tracked changes' in Word files as these are lost in PDF conversion. I should be grateful if you would also provide a point-by-point response detailing

© 2021. Published by The Company of Biologists under the terms of the Creative Commons Attribution License (https://creativecommons.org/licenses/by/4.0/).

how you have dealt with the points raised by the reviewers in the 'Response to Reviewers' box. If you do not agree with any of their criticisms or suggestions please explain clearly why this is so.

Reviewer 1

Advance summary and potential significance to field

Based on theoretical considerations this paper makes the point that elongation bias of early lung and kidney branches may perhaps be attributed to shear stress signaling on the apical surface of the epithelia. The authors include experiments confirming that mesenchyme per se is dispensible for this elongation and show that FGF signaling is likewise dispensable, although as they printout other growth factors are likely involved.

Comments for the author

To quote their own discussion summary:

"we have shown that flow- induced shear stress can, in principle, explain the biased elongating outgrowth of epithelial tubes in early lung and kidney development. Quantitative measurements of the flow velocity are now important to further validate this hypothesis. Moreover, it will be important to understand why the epithelial tubes collapse, and how the mesenchyme modulates the elongation bias".

For their assertions to stand on solid ground in Development, these measurements should be done and included in the paper.

Reviewer 2

Advance summary and potential significance to field

The authors claim to have eliminated various possible explanations for biased elongation in branching and to have identified a likely mechanism.

Alas I do not think they have quite done any of these things.

Comments for the author

Major concerns:

This is a very odd paper, especially given the excellence that I normally associate with the Iber lab. It poses an interesting question - why is growth of lung and kidney branches biased to elongation? That question is well within the area of interest of Development. But it then proceeds to try to eliminate various possibilities, with generally inconclusive small experiments (inconclusive because they involve too many untested assumptions - see below), and then presents a computer model of sheer stress based on a number of untested assumptions (see below) and, because the model produces nice results, pushes the idea that sheer stress is the mechanism (with no proper biological follow-up to test predictions from the model).

This seems a bad fit for Development. I think it would be entirely reasonable for the group to publish the sheer stress idea as a purely modelling paper, in a theoretically-orientated journal, as long as they deal honestly with the assumptions. The practical measurements near the beginning could easily be a small paper in a journal such as J Anat. The wet lab experiments in between are really not sound enough (because of the assumptions made) to fit anywhere.

Detailed comments:

Title: the title seems to apply to epithelial tubes generally. The paper is about one type of branching in two organ (mainly lung, some kidney). The title should reflect the precise scope of the

© 2021. Published by The Company of Biologists under the terms of the Creative Commons Attribution License (https://creativecommons.org/licenses/by/4.0/).

paper. By all means speculate in the discussion about generalizability, but don't claim it in the title.

Descriptive results (lines 109-133) are done very well and this is the section I mean above, that could go to a journal that likes anatomical description.

The next section is intended to show that mesenchyme is not needed for biased elongation. As a minor point, the authors should acknowledge prior work that showed this qualitatively (eg Sanjay Nigam's Matrige/ Collagen I culture of ureteric bud). The current MS is the first I know to use a quantitative method, though. It is critical that you separate demonstrating that active cell behaviours of mesenchyme are not needed (Nigam and you show this) from demonstrating that the matrix that mesenchyme makes is not needed. You grow these systems in a matrix and you really need to mention the presence of this in the results section (I had to dig through M&M to find this critical piece of info). I imagine you do not get branching without it, showing that it is critical (if I am wrong, and you do get branching without it, why have it there at all?).

Lines 160-163 describe experiments that contain hidden assumptions. To begin with, you assert that FGF/ GDNF are homogenous. How do you know that? Activities of the cells themselves can internalize these factors, or might destroy them locally - all you know is that you applied them in free solution, into a system in which diffusion is quite constrained by the gel. Second, while I enjoyed your earlier Turing papers, your claim in this current MS that GDNF/ FGF signalling must be concentrated at branch points does not follow from what you present here; GDNF/FGF signalling may be necessary to allow branching, but elevated signalling by them may not be present where branches emerge {Erk can be elevated by all kinds of things}. If you want to make this claim here, you need to tell the reader on what data it rests.

Lines 164-168: I take your point about absence of extensions, but can you rule out pulling by matrix contraction (eg tip cells release enzymes to cause this)? This is one source of pulling that would not have an obvious anatomical correlate from the cells. You might be able to get experiental evidence by cutting the matrix just ahead of a tip and seeing if the cut edge moves towards the tip (it was in tension) or remains closed (compression or neutral).

Lines 252-263: Assuming fluid flow (see below), you try to eliminate a direct pressure model by comparing growth of lungs with or without a trachea, to make drainage of the alleged fluid happen at different rates (around line 259). You see no difference, which you interpret as meaning that pressure is not relevant. This again rests on a number of assumptions, including that the flow exists, that if it does the trachea is a significant impediment to flow, and that the cutting (both the original dissection and removal of the trachea) does not simply block the tube in both cases. There are too many untested assumptions for this to be a helpful experiment.

The sheer stress section (mainly 266 onwards, but elements before it too): the whole validity of this rests on fluid being secreted into the lumen by the cells of the branches at this age. You cite (line 222) two sources to support your claim that this secretion exists; George et al. (2015) and Nelson et al. (2017). Neither of these sources provides any direct evidence for fluid secretion! The George paper is itself based entirely on modelling; like the manuscript on which I am commenting now, it assumes fluid secretion. The Nelson paper is experimental but does not provide any evidence for fluid secretion - it provides evidence that if one artificially creates a pressure gradient between lumenal and pleural cavities (lumenal higher), then growth and branching of a 12 day mouse lung tracheal system is enhanced over the 72h of the study. It provides no direct evidence for fluid secretion at this stage of development. And the fluid reported by Bokka et al. (cited in connection with viscosity in line 231) seems to have been taken from a prenatal mouse lung, not the really young kind being studied in this manuscript. Your model is fine, but should be either in a clearly modelling paper, or have at least some of its assumptions or predictions tested. How about measuring flow to show it is there (for example, microinject a fluorescent tracer in the lumen, photobleach one little area, and see whether the photobleached area moves in a particular direction or just diffuses). How about exposing cultured embryonic lung cells to sheer stress and seeing if you bias their division direction?

To conclude, while there is interesting material here, I felt I was reading three papers; a good anatomically descriptive one, a weak one of a few experimental tests, and a good purely modelling

© 2021. Published by The Company of Biologists under the terms of the Creative Commons Attribution License (https://creativecommons.org/licenses/by/4.0/).

one that could appear as such or be coupled with meaningful tests of its assumptions and predictions. Sorry for not being able to be more positive about this manuscript as it stands.

Reviewer 3

Advance summary and potential significance to field

In this study, Runser et al investigate lung and kidney morphogenesis. During morphogenesis, both organs contain tubular epithelial structures which lengthen faster than they grow in circumference, i.e. tissue growth in these tubes is locally anisotropic. The authors investigate the source of this anisotropy and put forward the hypothesis that cells use cilia to identify the direction of a constant fluid flow inside the tube, and that this signal provides a sufficient directional cue to guide tube elongation.

The authors first quantify the extent of tube elongation in lung and kidney morphogenesis. They then test a range of possible mechanisms that may impose growth directionality, and convincingly show that these mechanisms cannot be the source of tube elongation. Specifically, the authors exclude potential roles of the surrounding mesenchyme, of biased morphogen (FGF) signalling, of mechanical 'pulling' at branching points, and of pressure gradients of tubular fluid along the length of the tubes. As part of this investigation, the authors highlight that tubes are not circular, but flattened in cross-section. They computationally test a range of hypotheses that can contribute to this flattening, and show that stresses arising from this tube collapse dissipate quickly. Finally, the authors segment the precise shape of the inner tube shape of a lung epithelium, and simulate the fluid flow inside this segment. The model predicts that the arising shear forces are well within the regime that cilia are known to be able to sense showing that shear forces would indeed be able to provide a directional cue for tube elongation. This observation is linked with previous reports that genetic perturbations of cilia prevents tube elongation, in order to conclude that shear stresses must be the guiding factor in lung and kidney epithelial tube elongation.
Finally, a vertex model of anisotropic and longitudinally uniform growth is able to simultaneously account for previously reported cell shapes and cell division angles in lung epithelia, highlighting the fact that once isotropy is broken further known properties of the tissue naturally emerge.

This is a well-written paper with convincing experimental and in silico results that I believe will make a strong contribution to the Development Journal. In particular, the results appear generally applicable, since the experiments cover two organs, the mouse lung and kidney. As a particular strength, the authors combine results from embryo tissue dissection, as well as ex-vivo culture for both tissues. The experiments appear to me to be well-designed, in particular the removal of the mesenchyme to test its involvement in tube elongation, or the application of uniform growth factor concentrations reminded me of 'classical' embryology experiments. These experimental results are supported by three different computational models, which are each designed to answer specific questions.

Comments for the author

I believe a few minor revisions would be required before the final publication of this manuscript.

1.) In the section 'Mechanically forced tube collapse does not result in directional cues for uniform biased outgrowth': I agree that the presented model is very useful to illustrate the different ways in which this tube collapse could theoretically happen, and to highlight the fact that tensions can arise that will need to be dissipated by the tissue. However, I do not understand how the model results show that the tube collapse does not result in directional cues for tube elongation. What result could this model have given that would have informed us about possible directional cues? In order to provide insights on directional differences, wouldn't the model need to show that something is different in the length-direction of the tube ('out of the paper' in your images) than in the cross-sectional direction? Since the model only describes one of the two directions, I do not think it can tell us anything about such directional differences. In that sense the model is not fit-for-purpose. Please clarify if I am missing something here! I do not think this is a big issue, since the model does illuminate other interesting aspects of the tube collapse, so maybe this just needs to be described differently. Wouldn't the argument against tube collapse providing directional cues

© 2021. Published by The Company of Biologists under the terms of the Creative Commons Attribution License (https://creativecommons.org/licenses/by/4.0/).

be that the tube collapse is ‘the same’ along the length of the tube, both mechanically and topologically?

2.) In the same section, you use actin density as a read-out for tissue tension.
Doesn’t the actin density only show tensions that the tissue self-generates rather than tensions that arise from external pulling or compression? Hence, a tissue that is bent or compressed by external forces, like in your tube collapse model, might not contain localised actin. Or am I misunderstanding this?

3.) In the abstract you say: We use a cell-based vertex model to confirm that apical shear forces, unlike constricting forces, can give rise to … .
This makes it sound like there is a shear force that physically elongates cells directly, and that this is consistent with cell shape etc, but I don’t think that’s what you say in the paper. Maybe this should be ‘apical extension in response to shear forces’ or something like this, to be more accurate? Similar statements that may be misinterpreted appear in the main text. For example, you say ‘In a final step, we investigated whether shear stress, which results in a biased force in the longitudinal direction, could recapitulate the observed bias in apical cell shape, …’ Again this could easily be interpreted as apical domains being physically stretched by a shear force they experience.
4.) In figure 4B, you describe that ‘Isosurfaces highlight shape changes and volume increase over time’. This refers to the gray and blue boundary bits around the green signal, right? After staring at the figure for a while, I still cannot see how to interpret the visualisation so that I can tell the shape changes and volume increases. Is there a way to make this clearer, maybe with added descriptions or arrows?

5.) The computational results in the paper are not currently presented in a way that they can be repeated by someone who reads the paper, and this will need to be improved before publication. Examples below.

(i) It is not clear from the methods section how the finite-element model for tube collapse was implemented. Specifically, it is unclear what assumptions went into the equation of motion for this simulation, such as the stress-strain relationship for the material. Further, the text in the main manuscript states: ‘The tubular epithelial tissue was represented by an isotropic, linearly viscoelastic continuum’. This makes sense and it sounds like the ‘right’ thing to do here. However, the framework cited for this is for the ‘simulation of dense wire packings’, and according to the methods section the epithelium was represented by ‘third-order finite beam elements’. These three descriptions differ from each other. From the manuscript as it is currently written, I cannot see how these descriptions correspond to each other and fit together as a whole. In the revision, I think it needs to be clarified in which sense the epithelial tube corresponds to a wire packing, and how this analogy was used to arrive at the presented results. Ideally, it would be best to explicitly write out the equations of motion that were solved for Figure 5, and to add a brief description on the methods for numerical solution. Without this, it is difficult to properly evaluate this section as a reviewer (although it does appear to make sense in the main text and figure). In this model, it is also unclear how parameter values are chosen so that the simulations reflect viscoelastic properties of the tissue.

ii) In the vertex model, although the description is clearer than in the model for tube collapse, important details are also missing. For example, boundary conditions are not mentioned, it is not clear how forces were applied to boundary cells, what the magnitudes of the ‘pulling’ forces are, or what aspect ratio was assumed for the initial tissue configuration. For reproducibility, such details would need to be included.

iii) Some journals require source code for computational results to be made openly available. I do not know whether Development has a policy on this, but I would recommend to add the simulation code for the various simulations to the paper, for example as supplementary material or as links to github repositories.

© 2021. Published by The Company of Biologists under the terms of the Creative Commons Attribution License (https://creativecommons.org/licenses/by/4.0/).

First revision

Author response to reviewers' comments

Response to Reviewers

Reviewer 1 Advance summary and potential significance to field
Based on theoretical considerations this paper makes the point that elongation bias of early lung and kidney branches may perhaps be attributed to shear stress signaling on the apical surface of the epithelia. The authors include experiments confirming that mesenchyme per se is dispensible for this elongation and show that FGF signaling is likewise dispensable, although as they printout other growth factors are likely involved.

Reviewer 1 Comments for the author
To quote their own discussion summary: "we have shown that flow-induced shear stress can, in principle, explain the biased elongating outgrowth of epithelial tubes in early lung and kidney development. Quantitative measurements of the flow velocity are now important to further validate this hypothesis. Moreover, it will be important to understand why the epithelial tubes collapse, and how the mesenchyme modulates the elongation bias".

For their assertions to stand on solid ground in Development, these measurements should be done and included in the paper.

In the paper, we present a lot of experimental data, theory and computational studies to rule out current proposals of how epithelial tubes elongate during lung and kidney development. The key prediction of the paper is that the elongation bias is driven by fluid flow. Fluid flow has only been shown so far at E12.5 (Figure 3F and Movie 2 in Nelson et al., 2017, Development), but not at the earlier developmental stages. Given the importance of this prediction, we agree with the referee that it is important to test this. We had hoped to leave this to experimental groups, but given the request by this referee and referee 2, we have now spent a lot of effort to test it ourselves. To this end, we have developed a protocol, where we inject and follow bead movement in cultured lung explants. We are delighted to say that the experiments confirm our predictions. We hope that the new data now convinces the referee.

In the manuscript, we also evaluated several candidate mechanisms that can lead to tube collapse, and showed that none of these mechanisms would impact on the bias of elongating outgrowth. As the mechanisms that lead to tube collapse are thus unlikely do explain biased elongation (beyond the resulting smaller luminal space that increases the shear stress), their delineation seems to us peripheral in the context of this paper, and we would therefore like to leave this highly interesting problem to future studies.

Similarly, we show that the mesenchyme modulates the elongation bias, but is not necessary for biased elongating outgrowth. As this paper here focuses on the driver of biased elongating outgrowth, the impact of the mesenchyme is peripheral to this topic, and we would like to leave it to future studies.

Reviewer 2 Advance summary and potential significance to field
The authors claim to have eliminated various possible explanations for biased elongation in branching and to have identified a likely mechanism.

Alas I do not think they have quite done any of these things.

Reviewer 2 Comments for the author

Major concerns:

This is a very odd paper, especially given the excellence that I normally associate with the Iber lab. It poses an interesting question - why is growth of lung and kidney branches biased to elongation? That question is well within the area of interest of Development. But it then proceeds to try to eliminate various possibilities, with generally inconclusive small experiments (inconclusive because

© 2021. Published by The Company of Biologists under the terms of the Creative Commons Attribution License (https://creativecommons.org/licenses/by/4.0/).

they involve too many untested assumptions - see below), and then presents a computer model of sheer stress based on a number of untested assumptions (see below) and, because the model produces nice results, pushes the idea that sheer stress is the mechanism (with no proper biological follow-up to test predictions from the model).

This seems a bad fit for Development. I think it would be entirely reasonable for the group to publish the sheer stress idea as a purely modelling paper, in a theoretically-orientated journal, as long as they deal honestly with the assumptions. The practical measurements near the beginning could easily be a small paper in a journal such as J Anat. The wet lab experiments in between are really not sound enough (because of the assumptions made) to fit anywhere.

All experiments and theoretical studies are geared towards the same question: what drives the biased elongating outgrowth of epithelial tubes in developing lungs and kidneys? As such, we would not want to split the work on several papers, even if we agree that an incredible amount of work has gone into this single publication.

We chose Development as a journal because we had hoped that the paper would inspire experimental groups to test the key prediction of fluid flow in early lung development, but given the request by this referee and referee 1, we have now spent a lot of effort to test it ourselves. To this end, we have developed a protocol, where we inject and follow bead movement in the cultured lung explants. We are delighted to say that the experiments confirm our predictions. In addition, we have also tested the impact of matrix remodelling, and we show that while it affects growth and branching (as had been reported before), it does not affect the elongation bias. We address the additional concerns that the referee voiced in detail below, and we hope that the referee will now agree with us that the work is of the same high standards as always.

Detailed comments:

Title: the title seems to apply to epithelial tubes generally. The paper is about one type of branching in two organ (mainly lung, some kidney). The title should reflect the precise scope of the paper. By all means speculate in the discussion about generalizability, but don't claim it in the title.

We had aimed for a succinct title, but to avoid any misconceptions, we have now changed this to "The Biomechanical Basis of Biased Epithelial Tube Elongation in Lung and Kidney Development".

Descriptive results (lines 109-133) are done very well and this is the section I mean above, that could go to a journal that likes anatomical description.

We are glad the referee liked the work. We emphasize again that all experiments and theoretical studies in this paper are geared towards the same question: what drives the biased elongating outgrowth of epithelial tubes in developing lungs and kidneys? As such, we would not want to split the work on several papers, even if we agree that an incredible amount of work has gone into this single publication. The first figure is important to extend the previous seminal observations by Tang and co-workers to more developmental time points, and to show that the 2D cultures of lung rudiments recapitulate the in vivo situation. This lays the foundation for our 2D culture experiments in the following sections.

The next section is intended to show that mesenchyme is not needed for biased elongation. As a minor point, the authors should acknowledge prior work that showed this qualitatively (eg Sanjay Nigam's Matrige/ Collagen I culture of ureteric bud). The current MS is the first I know to use a quantitative method, though.

Thank you for pointing out this missing reference, which we have now included together with similar references for the lung..

It is critical that you separate demonstrating that active cell behaviours of mesenchyme are not needed (Nigam and you show this) from demonstrating that the matrix that mesenchyme makes is not needed. You grow these systems in a matrix and you really need to mention the presence of this in the results section (I had to dig through M&M to find this critical piece of info). I imagine you

© 2021. Published by The Company of Biologists under the terms of the Creative Commons Attribution License (https://creativecommons.org/licenses/by/4.0/).

do not get branching without it, showing that it is critical (if I am wrong, and you do get branching without it, why have it there at all?).

This seems to be a misunderstanding. First of all, this paper is not about branching, but about the mechanism behind the biased outgrowth of epithelial tubes (i.e. the branches). In using matrigel, we follow a standard protocol, and indeed the epithelium would not survive when cultured in solution, and would also not grow when supplemented with a growth factor sufficient for epithelial growth when embedded in Matrigel, as shown for isolated lung epithelium grown on a transwell filter without addition of ECM gel (Nogawa and Ito, 1995). As this is a standard well-established protocol that has been used by colleagues in the field for decades, it felt natural to us to describe this in the methods section. From the way the referee phrased her/his comments, it sounds as if the referee feels that we are hiding an important detail. This was certainly not our intent when describing the methods in the Methods section, and we have added this information to the results section and the figure legend. We note that the endogenous extracellular matrix (ECM) is produced by both the epithelium and the mesenchyme, and is thus still present when we culture the epithelium in Matrigel. In addition, the epithelium secretes metalloproteases that modulate the ECM as we discuss in more details below.

Lines 160-163 describe experiments that contain hidden assumptions. To begin with, you assert that FGF/ GDNF are homogenous. How do you know that? Activities of the cells themselves can internalize these factors, or might destroy them locally - all you know is that you applied them in free solution, into a system in which diffusion is quite constrained by the gel.

We have now changed this to "uniformly added". But we should emphasize that measurements show that diffusion is rather fast in Matrigel, if not as fast as in aqueous solutions.

Second, while I enjoyed your earlier Turing papers, your claim in this current MS that GDNF/ FGF signalling must be concentrated at branch points does not follow from what you present here; GDNF/FGF signalling may be necessary to allow branching, but elevated signalling by them may not be present where branches emerge {Erk can be elevated by all kinds of things}. If you want to make this claim here, you need to tell the reader on what data it rests.

The point that we are making in the manuscript is that DESPITE the clear tip concentration of phosphorylated ERK, an established signalling factor downstream of FGF/GDNF, inhibition of FGF/GDNF signalling with SU5402 does not reduce the elongation bias. This means that something different than elevated FGF/GDNF signalling must drive the tube elongation bias. We have now rewritten this section and the abstract in a way that it is hopefully clearer and less contentious.

Lines 164-168: I take your point about absence of extensions, but can you rule out pulling by matrix contraction (eg tip cells release enzymes to cause this)? This is one source of pulling that would not have an obvious anatomical correlate from the cells. You might be able to get experiental evidence by cutting the matrix just ahead of a tip and seeing if the cut edge moves towards the tip (it was in tension) or remains closed (compression or neutral).

We now include an additional experiment with GM6001, which inhibits metalloproteases that degrade extracellular matrix ahead of the lung tip, and which has previously been shown to affect lung tube elongation and branching (Gill et al., 2006, Developmental Biology). While we confirm previous observations, we do not observe any impact on the bias of tube elongation (Figure 3).

Lines 252-263: Assuming fluid flow (see below), you try to eliminate a direct pressure model by comparing growth of lungs with or without a trachea, to make drainage of the alleged fluid happen at different rates (around line 259). You see no difference, which you interpret as meaning that pressure is not relevant. This again rests on a number of assumptions, including that the flow exists, that if it does the trachea is a significant impediment to flow, and that the cutting (both the original dissection and removal of the trachea) does not simply block the tube in both cases. There are too many untested assumptions for this to be a helpful experiment.

As we pointed out in the discussion, we do agree that the demonstration of fluid flow in early stages of lung development is a key test of our theory. We had previously hoped to leave this difficult experiment to experimental groups, but in response to the comments by you and referee

© 2021. Published by The Company of Biologists under the terms of the Creative Commons Attribution License (https://creativecommons.org/licenses/by/4.0/).

1, we have now spent months to experimentally test this. We are delighted to say that the experiments confirm our prediction.

As part of the experiment that demonstrates the existence of flow, we now also demonstrate outflow from the trachea. Cutting the trachea thus does not block outflow. Earlier experiments already demonstrated that the tube must be actively sealed to block outflow, and blocking outflow then results in an increase in pressure and concomitant shape changes (Blewett et al., 1996; Nelson et al., 2017; Unbekandt et al., 2008). Such shape change is not observed when we cut the trachea.

Given the existence of flow, there must be a pressure gradient. While we cannot measure the exact shape of the pressure gradient, the pressure must be higher at the tips than at the larynx to generate an outflow. If pressure impacted directly on the tube shape, then the tubes would have to be wider the longer the distance from the outlet. We do not observe this. This argues against a substantial fluid-structure interaction.

The sheer stress section (mainly 266 onwards, but elements before it too): the whole validity of this rests on fluid being secreted into the lumen by the cells of the branches at this age. You cite (line 222) two sources to support your claim that this secretion exists; George et al. (2015) and Nelson et al. (2017). Neither of these sources provides any direct evidence for fluid secretion! The George paper is itself based entirely on modelling; like the manuscript on which I am commenting now, it assumes fluid secretion. The Nelson paper is experimental but does not provide any evidence for fluid secretion - it provides evidence that if one artificially creates a pressure gradient between lumenal and pleural cavities (lumenal higher), then growth and branching of a 12 day mouse lung tracheal system is enhanced over the 72h of the study. It provides no direct evidence for fluid secretion at this stage of development. And the fluid reported by Bokka et al. (cited in connection with viscosity in line 231) seems to have been taken from a prenatal mouse lung, not the really young kind being studied in this manuscript. Your model is fine, but should be either in a clearly modelling paper, or have at least some of its assumptions or predictions tested. How about measuring flow to show it is there (for example, microinject a fluorescent tracer in the lumen, photobleach one little area, and see whether the photobleached area moves in a particular direction or just diffuses). How about exposing cultured embryonic lung cells to sheer stress and seeing if you bias their division direction?

A movie in the Supplementary Material of Nelson et al., 2017, Development demonstrates fluid flow in mouse E12.5 lung rudiments. However, as we pointed out in the discussion, we fully agree that the demonstration of fluid flow before E12.5 is key to confirm our theory. We had previously hoped to leave this difficult experiment to experimental groups, but in response to the comments by you and referee 1, we have now spent months to experimentally test this. We are delighted to say that the experiments confirm our prediction. We emphasize that our shear stress mechanism requires flow, but how the flow arises is not important. As such, it is not relevant for us to demonstrate fluid secretion. We have rephrased the sections accordingly.

Unfortunately, it is not possible to do the suggested measurements to show that shear stress alters the apical cell shape in developing mouse lungs because the apical cell shapes cannot be imaged in live lung explants with currently available technologies, to the best of our knowledge. It would only be possible to isolate individual epithelial cells, fix them to a microfluidic chip and apply a fluid flow. However, in that case, they are not retaining the 3D cell shape that they have in epithelial tubes, and it is thus impossible to evaluate the impact of the fluid flow on the apical cell shape. As we note, experiments in a wide range of epithelial systems (where Calcium is used as readout) already demonstrate the responsiveness of epithelial cells to shear stress, and we therefore refer the reader to the existing literature. We hope that our paper will encourage the experiment that you suggest once technology becomes available to do this type of experiment.

To conclude, while there is interesting material here, I felt I was reading three papers; a good anatomically descriptive one, a weak one of a few experimental tests, and a good purely modelling one that could appear as such or be coupled with meaningful tests of its assumptions and predictions. Sorry for not being able to be more positive about this manuscript as it stands.

© 2021. Published by The Company of Biologists under the terms of the Creative Commons Attribution License (https://creativecommons.org/licenses/by/4.0/).

We agree that we included a lot of work in a single paper, but all contribute to a single question: what drives biased elongation of epithelial tubes in developing lungs and kidneys? In the paper, we show

1. That the lung and kidney epithelial tubes undergo biased elongating outgrowth in early development
2. That neither the mesenchyme, nor FGF/GDNF signalling, nor localised ECM turn-over are necessary for the biased outgrowth.
3. That the epithelial tubes are collapsed
4. That fluid flow is present already in early lung development, and that the observed flow in the collapsed tubes results in shear stress levels that epithelial cells are known to be able to sense
5. That a tangential force as generated by shear stress results in elongation and cell shape bias as previously measured

We hope that the story line is now clearer to the referee, and she / he will now endorse our manuscript.

Reviewer 3 Advance summary and potential significance to field
In this study, Runser et al investigate lung and kidney morphogenesis. During morphogenesis, both organs contain tubular epithelial structures which lengthen faster than they grow in circumference, i.e. tissue growth in these tubes is locally anisotropic. The authors investigate the source of this anisotropy and put forward the hypothesis that cells use cilia to identify the direction of a constant fluid flow inside the tube, and that this signal provides a sufficient directional cue to guide tube elongation.

The authors first quantify the extent of tube elongation in lung and kidney morphogenesis. They then test a range of possible mechanisms that may impose growth directionality, and convincingly show that these mechanisms cannot be the source of tube elongation. Specifically, the authors exclude potential roles of the surrounding mesenchyme, of biased morphogen (FGF) signalling, of mechanical 'pulling' at branching points, and of pressure gradients of tubular fluid along the length of the tubes. As part of this investigation, the authors highlight that tubes are not circular, but flattened in cross-section. They computationally test a range of hypotheses that can contribute to this flattening, and show that stresses arising from this tube collapse dissipate quickly. Finally, the authors segment the precise shape of the inner tube shape of a lung epithelium, and simulate the fluid flow inside this segment. The model predicts that the arising shear forces are well within the regime that cilia are known to be able to sense, showing that shear forces would indeed be able to provide a directional cue for tube elongation. This observation is linked with previous reports that genetic perturbations of cilia prevents tube elongation, in order to conclude that shear stresses must be the guiding factor in lung and kidney epithelial tube elongation. Finally, a vertex model of anisotropic and longitudinally uniform growth is able to simultaneously account for previously reported cell shapes and cell division angles in lung epithelia, highlighting the fact that once isotropy is broken, further known properties of the tissue naturally emerge.

This is a well-written paper with convincing experimental and in silico results that I believe will make a strong contribution to the Development Journal. In particular, the results appear generally applicable, since the experiments cover two organs, the mouse lung and kidney. As a particular strength, the authors combine results from embryo tissue dissection, as well as ex-vivo culture for both tissues. The experiments appear to me to be well-designed, in particular the removal of the mesenchyme to test its involvement in tube elongation, or the application of uniform growth factor concentrations reminded me of 'classical' embryology experiments. These experimental results are supported by three different computational models, which are each designed to answer specific questions.

Thank you.

Reviewer 3 Comments for the author
I believe a few minor revisions would be required before the final publication of this manuscript.

1.) In the section 'Mechanically forced tube collapse does not result in directional cues for uniform biased outgrowth': I agree that the presented model is very useful to illustrate the different ways

© 2021. Published by The Company of Biologists under the terms of the Creative Commons Attribution License (https://creativecommons.org/licenses/by/4.0/).

in which this tube collapse could theoretically happen, and to highlight the fact that tensions can arise that will need to be dissipated by the tissue. However, I do not understand how the model results show that the tube collapse does not result in directional cues for tube elongation. What result could this model have given that would have informed us about possible directional cues? In order to provide insights on directional differences, wouldn't the model need to show that something is different in the length-direction of the tube ('out of the paper' in your images) than in the cross-sectional direction? Since the model only describes one of the two directions, I do not think it can tell us anything about such directional differences. In that sense the model is not fit-for-purpose. Please clarify if I am missing something here! I do not think this is a big issue, since the model does illuminate other interesting aspects of the tube collapse, so maybe this just needs to be described differently. Wouldn't the argument against tube collapse providing directional cues be that the tube collapse is 'the same' along the length of the tube, both mechanically and topologically?

We thank the reviewer for bringing this to our attention. These aspects deserve a clearer explanation indeed.

Aside from illustrating the different mechanical mechanisms that could lead to the observed tube collapse, the purpose of these simulations is to demonstrate mainly that all three scenarios give rise to energetic and geometric patterns that are highly non-uniform along the circumference of the tube. For biased outgrowth to be as uniform as observed experimentally, i.e., with approximately the same axial tissue growth rate and the same cell shape bias all around the circumference, any driving force for straight outgrowth would be expected to act relatively uniformly along the circumference. Without such uniformity, biased outgrowth would lead to curving of growing tubular segments or other distortions in the growth pattern, also on the cellular level, which are not generally observed. If stress or curvature cues from tube collapse were responsible for biased outgrowth, it would lead to non-uniform growth, because those cues are very non-uniformly distributed along the circumference, as shown by our simulations.

We have chosen to perform these simulations in two-dimensional cross sections to exploit the symmetry present in the tubular segments of the epithelium. Aside from minor boundary effects, a full three-dimensional simulation would yield the same results as our 2D simulations extruded in the third direction (the tube length), thus providing no additional insight except that the simulation could have been performed in 2D. To answer the reviewer's specific question: The model would not necessarily need to show any differential along the length-direction of the tube to provide insight into directional growth bias. In fact it wouldn't either in an analogous 3D simulation. As the reviewer correctly noted, the tube collapse is the same along the length (up to boundary effects). That alone, however, does not rule out tube collapse as a source for directional cues, as there could still be a difference in axial and circumferential stress. What we are showcasing is the non-uniformity in circumferential direction, and the absence of any substantial stress or curvature in the majority of the circumference. If the resulting stress or curvature patterns had come out as more uniform from these simulations, a three-dimensional simulation would have been warranted to compare circumferential to axial effects, the latter arising from other mechanisms than the three we examined.

There is, however, a subtlety in the terminology that we may not have handled carefully enough in this context. When the stress and curvature patterns arising from collapse in our simulations are extruded in the third direction, they do form something that could be interpreted as a directional cue for biased outgrowth. In the cross section, both hoop stress and curvature are localised at two points: the two extremities of the collapsed cross section. Extruded along the tube axis, these point-like localisations form tangential lines in longitudinal direction, which could, in principle, provide the required directionality. However, for such localised lines to lead to uniform biased outgrowth, an intercellular communication mechanism would be required that distributes the directional cue from the two extremal lines to all cells on the circumference. Without such a mechanism, for which there is no indication, substantial distortions of the tissue on the cellular scale would be expected in the vicinity of the cue lines, which are not observed.

We have rephrased and extended the text in this section in the manuscript to discuss these aspects in greater detail and to improve its clarity.

© 2021. Published by The Company of Biologists under the terms of the Creative Commons Attribution License (https://creativecommons.org/licenses/by/4.0/).

2.) In the same section, you use actin density as a read-out for tissue tension. Doesn't the actin density only show tensions that the tissue self-generates, rather than tensions that arise from external pulling or compression? Hence, a tissue that is bent or compressed by external forces, like in your tube collapse model, might not contain localised actin. Or am I misunderstanding this?

Actin filaments were reported to be able to function as tension sensors [R1]. While there is also evidence for actin polymerization from external mechanical stimuli [R2,R3], we agree that it is not conclusively established that actin density increases in tissue regions that are externally compressed, stretched or bent. As such, we agree that the actin staining cannot rule out that tension patterns exist (among others in the ECM), especially as tension can be relaxed by remodelling. In that sense, our actin staining only provides no further evidence for tension, but does not conclusively exclude the possibility of residual stress. We have rephrased this paragraph in the manuscript to put this into perspective.

[R1] K.Hayakawa, H. Tatsumi, M. Sokabe, J Cell Biol (2011) 195 (5): 721-727
[R2] X. Shao, Q. Li, A. Mogilner, A. D. Bershadsky, G. V. Shivashankar, PNAS (2015), 112 (20): E2595-E2601
[R3] H. Hirata, H. Tatsumi, M. Sokabe, J. Cell Sci. (2008) 121: 2795-2804

3.) In the abstract you say: We use a cell-based vertex model to confirm that apical shear forces, unlike constricting forces, can give rise to … .This makes it sound like there is a shear force that physically elongates cells directly, and that this is consistent with cell shape etc, but I don't think that's what you say in the paper. Maybe this should be 'apical extension in response to shear forces' or something like this, to be more accurate? Similar statements that may be misinterpreted appear in the main text. For example, you say 'In a final step, we investigated whether shear stress, which results in a biased force in the longitudinal direction, could recapitulate the observed bias in apical cell shape, …' Again this could easily be interpreted as apical domains being physically stretched by a shear force they experience.

This is a very important point, and we have now added a paragraph to the main text to explain this in detail. We now stress that it does not matter for the outcome of the cell-based tissue model whether the shear force is exerted directly or indirectly via intracellular processes. As such, we would like to keep the abstract as is, as the important difference is only in whether the force is applied in longitudinal direction (shear stress) or in the orthogonal direction (constricting forces). Only in the former but not in the latter case, we can quantitatively match the data.

4.) In figure 4B, you describe that 'Isosurfaces highlight shape changes and volume increase over time'. This refers to the gray and blue boundary bits around the green signal, right? After staring at the figure for a while, I still cannot see how to interpret the visualisation so that I can tell the shape changes and
volume increases. Is there a way to make this clearer, maybe with added descriptions or arrows?

We have now rephrased this part of the legend to read "Iso-surface overlays highlight shape changes; domain volumes are given on the lower left."

5.) The computational results in the paper are not currently presented in a way that they can be repeated by someone who reads the paper, and this will need to be improved before publication. Examples below.

(i) It is not clear from the methods section how the finite-element model for tube collapse was implemented. Specifically, it is unclear what assumptions went into the equation of motion for this simulation, such as the stress-strain relationship for the material. Further, the text in the main manuscript states: 'The tubular epithelial tissue was represented by an isotropic, linearly viscoelastic continuum'. This makes sense and it sounds like the 'right' thing to do here. However, the framework cited for this is for the 'simulation of dense wire packings', and according to the methods section the epithelium was represented by 'third-order finite beam elements'. These three descriptions differ from each other. From the manuscript as it is currently written, I cannot see how these descriptions correspond to each other and fit together as a whole. In the revision, I think it needs to be clarified in which sense the epithelial tube corresponds to a wire packing, and how this analogy was used to arrive at the presented results. Ideally, it would be best to explicitly

© 2021. Published by The Company of Biologists under the terms of the Creative Commons Attribution License (https://creativecommons.org/licenses/by/4.0/).

write out the equations of motion that were solved for Figure 5, and to add a brief description on the methods for numerical solution. Without this, it is difficult to properly evaluate this section as a reviewer (although it does appear to make sense in the main text and figure). In this model, it is also unclear how parameter values are chosen so that the simulations reflect viscoelastic properties of the tissue.

We have extended the paragraph in the methods section on this subject to include all requested details, including the elastic energy that is minimized, Newton's second law which is solved, and all relevant simulation parameters. We emphasise, however, that all material parameters are not needed to reproduce our simulation results in Fig. 5 - a fact which may not be obvious to readers who are inexperienced with the subject. Since we solved for the quasi-static equilibrium solution of a simple enough geometry with linear elasticity, the employed mass density and viscous damping parameters are irrelevant. So is Young's modulus E, which is a mere prefactor to all elastic energies in the system, thus having no effect on the final equilibrium simulation result. Since the simulations are restricted to 2D and are based on classical beam theory, also the second elastic parameter, Poisson's ratio v, barely affects the outcome. The applied pressure in the pressure collapse scenario scales with the elastic modulus and is simply set large enough to just overcome the critical buckling pressure of the tube, making it also irrelevant. This leaves only the geometrical parameters to be specified. There are essentially two such parameters in the simulated system: the epithelial thickness t and the tube radius R. Only their ratio t/R=0.5, which is given in the main text, is relevant - choosing the other one of the two freely will yield the same simulation result.

We have also extended and unified the technical description of the implementation, which appears to have been unclear. The source that we refer to for details of the employed beam finite element framework, titled "Finite element simulation of dense wire packings" (Vetter et al., 2013) is the original publication that provides all technical details of our model, although the relationship may seem unclear from the title. The numerical framework was originally developed with general elastic structures (wires in that paper) in mind, but it is general enough to be applicable to 2D viscoelastic tissue simulations. There are therefore no three different descriptions, just one. We hope that with the substantially expanded description in the methods section, this will now be sufficiently evident.

ii) In the vertex model, although the description is clearer than in the model for tube collapse, important details are also missing. For example, boundary conditions are not mentioned, it is not clear how forces were applied to boundary cells, what the magnitudes of the 'pulling' forces are, or what aspect ratio was
assumed for the initial tissue configuration. For reproducibility, such details would need to be included.

We have edited the cell-based simulation paragraph to address the comments made by the reviewer.

iii) Some journals require source code for computational results to be made openly available. I do not know whether Development has a policy on this, but I would recommend to add the simulation code for the various simulations to the paper, for example as supplementary material or as links to github repositories.

We now provide the code for the COMSOL Multiphysics (Fig. 6) and Chaste (Fig. 7) simulations via a gitlab repository (link added to the manuscript). The simulations in Fig. 5 were carried out with a custom-made closed-source framework whose scope goes beyond the application presented in this paper. All implementation details are given in the cited source (Vetter et al., 2013) and will allow the gentle reader to fully reproduce the simulations.

© 2021. Published by The Company of Biologists under the terms of the Creative Commons Attribution License (https://creativecommons.org/licenses/by/4.0/).

Second decision letter

MS ID#: DEVELOP/2020/194209

MS TITLE: The Biomechanical Basis of Biased Epithelial Tube Elongation in Lung and Kidney Development

AUTHORS: Lisa Conrad, Steve Runser, Harold Gomez, Christine Lang, Mathilde Dumont, Aleksandra Sapala, Laura Kramps, Odysse Michos, Roman Vetter, and Dagmar Iber

I have now received all the referees reports on the above manuscript, and have reached a decision. The referees' comments are appended below, or you can access them online: please go to BenchPress and click on the 'Manuscripts with Decisions' queue in the Author Area.

The overall evaluation is very positive and we would like to publish a revised manuscript in Development. Two referees are making (minor) comments/suggestions and we would like to offer you the opportunity to address them. If you do not agree with any of their criticisms or suggestions explain clearly why this is so.

Reviewer 1

Advance summary and potential significance to field

This paper discusses the functional role of shear forces in epithelial tube elongation in the lung and kidney and supports the idea that these tube elongate at their tips due to growth factors signaling and matrix remodeling.

Comments for the author

This is a clever and detailed paper looking at the functional role of shear forces at the ides and tips in tube elongation comparing embryonic lung and kidney. They conclude that growth factors and matrix remodeling drive the elongation process, both ideas that are quite well established.
It would be interesting to know what the authors think about the scaling of tube length and the decision to branch stereotypically not only in the murine context but also across species with much larger airways and longer interbranch lengths like human.

Reviewer 2

Advance summary and potential significance to field

The authors have asked a question that people tend not to ask (how to tubes elongate rather than just be spheres) and have come up with an interesting tentative answer supported by modelling and by an important new wet lab experiment not in the original submission.

Comments for the author

The authors have responded to my suggestions - they have in fact done much more than I had imagined they would in order to justify a publication of this whole work rather than following my original suggestion of splitting to different types of journal. They have also been a lot clearer about what they are driving at, and I have no further suggestions for change.

I think this paper has the potential to become a classic - it will not pick up huge numbers of citations quickly but it will pick them up for many many years to come.

© 2021. Published by The Company of Biologists under the terms of the Creative Commons Attribution License (https://creativecommons.org/licenses/by/4.0/).

Reviewer 3

Advance summary and potential significance to field

The authors have convincingly addressed all of my comments. As outlined in my previous review, I believe that this is a beautiful paper and a good fit for Development. With the addition of the new experiments on fluid flow, the paper has now been strengthened further. I appreciate that simulations codes are now provided, and that the implementation of the computational models has been clarified.

Comments for the author

There is one minor remaining comment that the authors may wish to address at their own/ the editor's discretion. This comment is in relation to point 1 that I previously raised. I now understand the arguments on the tissue collapse better than before, and I find the revised manuscript has indeed been clarified in that section. However, I now slightly disagree with the statement "Mechanically forced tube collapse does not result in directional cues for uniform biased outgrowth".
Rather than 'does not', I think the statement should say 'is unlikely to'. Since the tube collapse does lead to lines of higher curvature oriented in the elongation direction at the 'bends' of the collapse, there is indeed a potential directional cue. The authors argue well that this cue is unlikely to direct tube elongation, but they do not have evidence to completely show that it is indeed irrelevant, and I do not know how one would even go about finding such evidence.

Second revision

Author response to reviewers' comments

Response to Reviewers

Reviewer 1 Advance summary and potential significance to field
This paper discusses the functional role of shear forces in epithelial tube elongation in the lung and kidney and supports the idea that these tube elongate at their tips due to growth factors signaling and matrix remodeling.

Reviewer 1 Comments for the author
This is a clever and detailed paper looking at the functional role of shear forces at the ides and tips in tube elongation comparing embryonic lung and kidney. They conclude that growth factors and matrix remodeling drive the elongation process, both ideas that are quite well established. It would be interesting to know what the authors think about the scaling of tube length and the decision to branch stereotypically not only in the murine context but also across species with much larger airways and longer interbranch lengths like human.

We thank the reviewer for the positive evaluation. The problem of stereotypic branching, branch distance, and the final remodelling into a fractal-like tree are distinct problems from the one of biased elongation that we consider here. As mentioned in the Introduction, we would argue that stereotypic branching is achieved via a ligand-receptor based Turing mechanism, which also sets the branch distance. We now added the last aspect in the Introduction. What drives the later remodelling is unknown and an interesting future research avenue, as we already mention in the discussion. Finally, the evolution of pattering mechanism is a very interesting point. So far, we have not seen 3D data from human embryonic lungs that one could use to work on this. In other systems, such as the neural tube, the patterning distances are not as different as the overall difference in embryo size may suggest. It will be very interesting to see whether this applies also to the lung and kidney.

© 2021. Published by The Company of Biologists under the terms of the Creative Commons Attribution License (https://creativecommons.org/licenses/by/4.0/).

Reviewer 2 Advance summary and potential significance to field
The authors have asked a question that people tend not to ask (how to tubes elongate rather than just be spheres) and have come up with an interesting tentative answer supported by modelling and by an important new wet lab experiment not in the original submission.

Reviewer 2 Comments for the author
The authors have responded to my suggestions - they have in fact done much more than I had imagined they would in order to justify a publication of this whole work rather than following my original suggestion of splitting to different types of journal. They have also been a lot clearer about what they are driving at, and I have no further suggestions for change.

I think this paper has the potential to become a classic - it will not pick up huge numbers of citations quickly but it will pick them up for many many years to come.

Thank you.

Reviewer 3 Advance summary and potential significance to field
The authors have convincingly addressed all of my comments. As outlined in my previous review, I believe that this is a beautiful paper and a good fit for Development. With the addition of the new experiments on fluid flow, the paper has now been strengthened further. I appreciate that simulations codes are now provided, and that the implementation of the computational models has been clarified.

Thank you.

Reviewer 3 Comments for the author
There is one minor remaining comment that the authors may wish to address at their own/ the editor's discretion. This comment is in relation to point 1 that I previously raised. I now understand the arguments on the tissue collapse better than before, and I find the revised manuscript has indeed been clarified in that section. However, I now slightly disagree with the statement “Mechanically forced tube collapse does not result in directional cues for uniform biased outgrowth”. Rather than ‘does not’, I think the statement should say ‘is unlikely to’. Since the tube collapse does lead to lines of higher curvature oriented in the elongation direction at the ‘bends’ of the collapse, there is indeed a potential directional cue. The authors argue well that this cue is unlikely to direct tube elongation, but they do not have evidence to completely show that it is indeed irrelevant, and I do not know how one would even go about finding such evidence.

We are happy to tone it down to “unlikely”.

<u>Third decision letter</u>

MS ID#: DEVELOP/2020/194209

MS TITLE: The Biomechanical Basis of Biased Epithelial Tube Elongation in Lung and Kidney Development

AUTHORS: Lisa Conrad, Steve Runser, Harold Fernando GÃ³mez, Christine Lang, Mathilde Dumond, Aleksandra Sapala, Laura Schaumann, Odysse Michos, Roman Vetter, and Dagmar Iber
ARTICLE TYPE: Research Article

I am happy to tell you that your manuscript has been accepted for publication in Development, pending our standard ethics checks.

© 2021. Published by The Company of Biologists under the terms of the Creative Commons Attribution License (https://creativecommons.org/licenses/by/4.0/).
